# Supplementary material for: Diverse Mechanisms of Antimicrobial Activities of Lactoferrins, Lactoferricins, and Other Lactoferrin-Derived Peptides
Source: Int J Mol Sci. 2021 Oct 19;22(20):11264. doi: 10.3390/ijms222011264 (PMC8541349; doi:10.3390/ijms222011264)
Supplement: Supplementary file 1 [file ijms-22-11264-s001.zip › ijms-1373357-supplementary.pdf]

Diverse mechanisms of antimicrobial activities of lactoferrins, lactoferricins, and other lactoferrin-derived peptides

Cell and molecular life science

Špela Gruden, Nataša Poklar Ulrih*

Department of Food Science and Technology, Biotechnical Faculty, University of Ljubljana, 1000 Ljubljana, Slovenia,

***Corresponding author: Nataša Poklar Ulrih**

Department of Food Science and Technology

Biotechnical Faculty

University of Ljubljana

Jamnikarjeva 101

1000 Ljubljana, Slovenia,

Tel: +386-1-3203 780

E-mail: natasa.poklar@bf.uni-lj.si

Code availability: Not applicable.

Supplementary Table S1. Studies carried out to date on bacteria, viruses, and fungi that are either susceptible or resistant (designated as R) to antimicrobial activity of lactoferrins, lactoferricins, and lactoferrin-derived (Lf peptide) and lactoferricin-derived (Lf-cin peptide) peptides.

Species	Source of lactoferrins used[1]					Peptide fraction[2]	References
	Lactoferrin	Lf peptide	Lactoferricin	Lf-cin peptide	Lf-ampin		
**Gram-positive bacteria**							
Bacillus cereus			Bovine			f(17-41)	[100]
		I: bovine				f(17-42)	[132]
Bacillus circulans			Bovine			f(17-41)	[100]
Bacillus natto			Bovine			f(17-41)	[100]
Bacillus subtilis			Bovine			f(17-41)	[100, 110]
				15: bovine		f(17-31)	[110, 114]
				11: bovine		f(20-30)	[110, 112]
				9: bovine (R)		f(20-28)	[110]
				6: bovine (R)		f(20-25)	[114]
					Bovine	f(268-284)	[139]
Bifidobacterium bifidum			Bovine (R)			f(17-41)	[100]
Bifidobacterium adolescentis			Bovine			f(17-41)	[100]
Bifidobacterium breve			Bovine			f(17-41)	[100]
Bifidobacterium longum			Bovine			f(17-41)	[100]

Bifidobacterium infantis			Bovine	f(17-41)	[100]
Clostridium perfrigens			Bovine	f(17-41)	[100]
Clostridium parapurtificum			Bovine	f(17-41)	[100]
Corynebacterium ammoniagenes			Bovine	f(17-41)	[100]
Corynebacterium diphtheriae			Bovine	f(17-41)	[100]
Corynebacterium renal			Bovine	f(17-41)	[100]
Enterococcus faecalis			Bovine (R)	f(17-41)	[100]
Lactobacillus casei	Human (R)			/	[71]
			Bovine	f(17-41)	[100]
Listeria monocytogenes	Bovine			/	[57, 135]
			Bovine	f(17-41)	[57, 100, 230]
		I: bovine		f(17-42)	[132]
		21LF: bovine		f(85-109)	[135]
		38LF: bovine		f(341-366)	[135]
		45LF: bovine		f(282-307)	[135]
Micrococcus flavus	Bovine			/	[231]
	Caprine			/	[231]
	Ovine			/	[231]
			Bovine	f(17-41)	[231]
			Caprine	f(14-42)	[231]
		2: bovine		f(1-11)-(17-47)	[133]
		4: bovine		f(1-16)-(45-48)	[133]
Staphylococcus aureus	Bovine			/	[57]

	Human (R)					/	[71]
			Bovine			f(17-41)	[57, 98, 100, 110, 111]
				15: bovine		f(17-31)	[110, 111]
				11: bovine		f(20-30)	[110, 113]
				9: bovine (R)		f(20-28)	[110]
			Human			f(1-49)	[98]
			Caprine			f(17-41)	[98, 100]
			Murine			f(17-41)	[98]
				P20: porcine		f(18-37)	[99]
		I: bovine				f(17-42)	[132]
Staphylococcus epidermidis	Human (R)					/	[71]
			Bovine			f(17-41)	[100]
Staphylococcus haemolyticus			Bovine			f(17-41)	[100]
Staphylococcus hominus			Bovine			f(17-41)	[100]
Streptococcus bovis			Bovine			f(17-41)	[100]
Streptococcus cremoris			Bovine			f(17-41)	[100]
Streptococcus lactis	Human (R)					/	[71]
			Bovine			f(17-41)	[100]
Streptococcus (mitior) mitis	Human					/	[71]
Streptococcus mutans	Human					/	[59, 60, 71, 72]
			Human			f(1-49)	[100]
					Bovine	f(268-284)	[139]

	Chimera: bovine					f(17-30)-(265-284)	[137]
Streptococcus pneumoniae	Bovine					/	[84]
	Human					/	[71]
				14: bovine		f(17-30)	[84]
					Bovine	f(268-284)	[84]
	Chimera: bovine					f(17-30)-(265-284)	[84]
Streptococcus pyogenes	Human (R)					/	[71]
	Bovine					/	[135]
		21LF: bovine				f(85-109)	[135]
		38LF: bovine				f(341-366)	[135]
		45LF:bovine				f(282-307)	[135]
Streptococcus thermophilus			Bovine			f(17-41)	[100]
Streptococcus salivarius	Human					/	[71]
Streptococcus sanguis					Human	f(269-285)	[141]
	Chimera: bovine					f(17-30)-(265-284)	[137]
**Gram-negative bacteria**							
Aggregatibacter actinomycetemcomitans	Human					/	[74]
Bacteroides distasonis			Bovine			f(17-41)	[100]
Bacteroides vulgatus			Bovine			f(17-41)	[100]
Burkholderia pseudomallei	Chimera: bovine					f(17-30)-(265-284)	[144]
Escherichia coli	Bovine					/	[57, 75, 135, 231]
	Human (some R)					/	[60, 64, 71]

Caprine				/	[231]
Ovine				/	[231]
		Bovine		f(17-41)	[57, 58, 61, 98, 100, 101, 106, 110, 111, 231]
			15: bovine	f(17-31)	[109-111, 114]
			B 11: bovine	f(20-30)	[110, 112, 113]
			9: bovine (R)	f(20-28)	[110]
			6: bovine (R)	f(20-25)	[109, 110, 114]
		Human		f(1-49)	[98]
			5s: human	f(21-31)	[109]
			6s: human	f(19-31)	[109]
		Caprine		f(14-42)	[98, 231]
		Murine		f(17-41)	[98]
			P20: porcine	f(18-37)	[99]
	I: bovine			f(17-42)	[132]
	II: bovine			f(1-16)-(43-48)	[132]
	III: bovine			f(1-42)-(43-48)	[132]
	1A: bovine			f(17-41)	[136]
	1B: bovine			f(17-42)	[136]
	2: bovine			f(17-43)	[136]
	3: bovine			f(1-16)-(17-48)	[136]
	21LF: bovine			f(85-109)	[135]

		38LF: bovine				f(341-366)	[135]
		45LF: bovine				f(282-307)	[135]
					Bovine	f(268-284)	[139]
					Bovine	f(259–284)	[140]
					Bovine	f(265–296)	[140]
					Bovine	f(265–284)	[140]
					Human (R)	f(269-285)	[141]
	Chimera: bovine					f(17-30)-(265-284)	[137]
Enterobacter cloacae	Human (R)					/	[71]
Campylobacter jejuni			Bovine			f(17-41)	[100]
Helicobaeter pylori	Bovine					/	[127]
	Human					/	[127]
			Bovine			f(17-41)	[127]
				14: bovine (R)		f(17-30)	[137]
					Bovine (R)	f(269-285)	[137]
	Chimera: bovine					f(17-30)-(265-284)	[137]
Legionella pneumophila	Human					/	[73]
Proteus mirabilis				B: bovine (R)		f(17-41)	[98]
Proteus vulgaris			Bovine			f(17-41)	[100]
Pseudomonas aeruginosa	Bovine (R)					/	[57]
	Human					/	[71]
			Bovine			f(17-41)	[57, 75, 100, 110]
				15: bovine		f(17-31)	[110]

				11: bovine		f(20-30)	[110]
				9: bovine (R)		f(20-28)	[110]
					Bovine	f(268-284)	[139]
	Chimera: bovine					f(17-30)-(265-284)	[137]
Pseudomonas fluorescens			Bovine (R)			f(17-41)	[100, 134]
		I: bovine				f(17-42)	[132]
		Bovine (R)				f(308-317)	[134]
Pseudomonas putida	Bovine					/	[75]
Pseudomonas syringae			Bovine			f(17-41)	[134]
		Bovine				f(308-317)	[134]
Salmonella choleraesuis			Bovine			f(17-41)	[110]
				15: bovine		f(17-31)	[110]
				11: bovine		f(20-30)	[110]
				9: bovine (R)		f(20-28)	[110]
Salmonella enteritidis			Bovine			f(17-41)	[100]
Salmonella montevideo			Bovine			f(17-41)	[61]
Salmonella newport	Human (R)					/	[71]
Salmonella salford		I: bovine				f(17-42)	[132]
Salmonella typhimurium	Human					/	[64]
			Bovine			f(17-41)	[61, 106, 110]
				15: bovine		f(17-31)	[110]
				11: bovine		f(20-30)	[110]
				9: bovine (R)		f(20-28)	[110]

Shigella sonnei	Human (R)					/	[71]
Shigella flexneri			Bovine			f(17-41)	[106]
Klebsiella pneumoniae	Bovine (R)					/	[57]
			Bovine			f(17-41)	[57, 100]
Vibrio cholerae	Bovine					/	[142, 232]
	Human					/	[60, 64, 71]
				14: bovine (R)		f(17-30)	[142]
					Bovine (R)	f(265-284)	[142]
	Chimera: bovine					f(17-30)-(265-284)	[142]
Vibrio parahaemolyticus	Bovine					/	[142]
				14: bovine (R)		f(17-30)	[142]
					Bovine (R)	f(265-284)	[142]
	Chimera: bovine					f(17-30)-(265-284)	[142]
Yersinia enterocolitica	Bovine					/	[135]
			Bovine (some R)			f(17-41)	[98, 100]
		21LF: bovine				f(85-109)	[135]
		38LF: bovine				f(341-366)	[135]
		45 LF: bovine				f(282-307)	[135]
				14: bovine		f(17-30)	[143]
					Bovine	f(265-284)	[143]
	Chimera: bovine					f(17-30)-(265-284)	[143]
Yersinia pseudotuberculosis				14: bovine (R)		f(17-30)	[143]
					Bovine (R)	f(265-284)	[143]

	Chimera: bovine		f(17-30)-(265-284)	[143]
**Enveloped viruses**				
Coronavirus				
SARS CoV (pseudovirus)	Bovine		/	[189]
SARS CoV 2	Human		/	[193, 194]
	Bovine		/	[191-193]
Feline calicivirus	Bovine		/	[171]
		Bovine	f(17-41)	[171]
Hantavirus	Bovine		/	[233]
Hepatitis B virus	Bovine		/	[151]
	Human		/	
Hepatitis C virus	Bovine		/	[150, 234, 235]
	Human		/	[150, 235]
		Bovine (R)	f(17-41)	[234]
		Bovine	f(600-632)	[180]
Hepatitis G virus	Bovine		/	[234]
Herpes simplex virus-1	Bovine		/	[146, 152, 154, 155, 173]
	Human		/	[146, 152, 154, 155]
	Caprine		/	[146]
		Bovine	f(17-41)	[146, 152]

			Human	f(1-49)	[146]
		19: bovine		f(86-258)	[182]
		28: bovine		f(345-689)	[182]
		30: bovine		f(1-280)	[182]
Herpes simplex virus-2	Bovine			/	[146, 236]
	Human			/	[146]
	Caprine			/	[146]
			Bovine	f(17-41)	[146]
			Human	f(1-49)	[146]
Human cytomegalovirus	Bovine			/	[154, 158, 159]
	Human			/	[154, 158, 159]
	Caprine			/	[158]
			Human	f(1-49)	[158]
Human immunodeficiency virus, type 1	Bovine			/	[159-162]
	Human			/	[159, 162]
			Bovine	f(17-41)	[160, 185]
Human immunodeficiency virus, type 2	Bovine			/	[162]
Influenza virus A (H1N1; H3N2)	Bovine			/	[147, 149]
		1: bovine		f(418-429)	[149]
		2: bovine		f(506-522)	[149]
		3: bovine		f(552-563) *	[149]

		14: bovine			f(426-429)	[178]
		15: bovine			f(422-425)	[178]
		17: bovine			f(418-421)	[178]
Respiratory syncytial virus	Human				/	[237]
**Naked viruses**						
Adenovirus	Bovine				/	[163-165]
	Human				/	[163, 164]
			Bovine		f(17-41)	[164]
Echovirus	Bovine				/	[148, 169]
			Bovine		f(17-41)	[148]
Enterovirus 71	Bovine				/	[238]
	Human				/	[238]
Human papillomavirus	Bovine				/	[166, 167]
	Human				/	[166, 167]
				15: bovine	f(17-31)	[167]
				16: bovine	f(17-42)	[167]
			Human		f(1-49)	[167]
Rotavirus	Bovine				/	[170, 198]
		Bovine			f(86-258)	[198]
		Bovine			f(324-329)	[198]
Poliovirus	Bovine				/	[168, 171]
			Bovine (R)		f(17-41)	[171]
**Yeast**						

Candida albicans	Bovine					/	[135]
	Human					/	[71, 199, 200]
			Bovine			f(17-41)	[98, 139, 202-204]
				14: bovine		f(17-30)	[139]
				15: bovine		f(17-31)	[114]
				6: bovine		f(20-25)	[114]
		2: bovine				f(17-26)	[205]
				P20: porcine		f(18-37)	[99]
		21LF: bovine				f(85-109)	[135]
		38LF: bovine				f(341-366)	[135]
		45LF: bovine				f(282-307)	[135]
					Bovine	f(268-284)	[139]
					Bovine	f(259–284)	[140]
					Bovine	f(265–296)	[140]
					Bovine	f(265–284)	[140]
					Human (R)	f(269-285)	[141]
Candida glabrata			Bovine (some R)			f(17-41)	[203]
Candida guilliermondii			Bovine			f(17-41)	[203]
Candida kefyr			Bovine			f(17-41)	[203]
Candida krusei	Human					/	[200]
			Bovine			f(17-41)	[203]
Candida parapsilois			Bovine			f(17-41)	[203]
Candida tropicalis			Bovine			f(17-41)	[98, 203]

Cryptococcus albidus	Bovine		f(17-41)	[204]
Cryptococcus curvatus	Bovine		f(17-41)	[204]
Cryptococcus neoformans	Bovine		f(17-41)	[98, 203]
Cryptococcus uniguttulatus	Bovine		f(17-41)	[204]
Saccharomyces cerevisiae	Bovine		f(17-41)	[203]
		15: bovine	f(17-31)	[114]
		6: bovine (R)	f(20-25)	[114]
Trichosporon cutaneum	Bovine		f(17-41)	[203, 204]
**Filamentous fungi**				
Absidia corymbifera	Bovine (some R)		f(17-41)	[203]
Aspergillus clavatus	Bovine (R)		f(17-41)	[203]
Aspergillus flavus	Bovine		f(17-41)	[203]
Aspergillus fumigatus	Bovine (some R)		f(17-41)	[203, 204]
Aspergillus nidulans		15: bovine	f(17-31)	[114]
		6: bovine	f(20-25)	[114]
Aspergillus niger	Bovine (some R)		f(17-41)	[203, 204]
Aspergillus versicolor	Bovine		f(17-41)	[203]
Botrytis cinerea		15:bovine	f(17-31)	[114]
		6: bovine	f(20-25)	[114]
Cladosporium trichoides	Bovine		f(17-41)	[203]
Epidermophyton floccosum	Bovine		f(17-41)	[203]
Exophiala dermatitidis	Bovine		f(17-41)	[203]
Fonsecaea pedrosoi	Bovine		f(17-41)	[203]

Fusarium moniliforme	Bovine		f(17-41)	[203]
Fusarium oxysporum		15: bovine	f(17-31)	[114]
		6: bovine	f(20-25)	[114]
Magnaporthe grisea		15: bovine (R)	f(17-31)	[114]
		6: bovine (R)	f(20-25)	[114]
Microsporum canis	Bovine		f(17-41)	[203]
Microsporum gypseum	Bovine		f(17-41)	[203]
Mucor circinelloides	Bovine (R)		f(17-41)	[203]
Mucor racemosus	Bovine		f(17-41)	[203]
Nannizzia gypsea	Bovine		f(17-41)	[204]
Nannizzia incurvata	Bovine		f(17-41)	[204]
Nannizzia otae	Bovine		f(17-41)	[204]
Paracoccidioides brasiliensis	Bovine		f(17-41)	[203]
Penicillium digitatum		Bovine	f(17-31)	[114]
		Bovine	f(20-25)	[114]
Penicillium expansum	Bovine (R)		f(17-41)	[203]
		15: bovine	f(17-31)	[114]
		6: bovine	f(20-25)	[114]
Penicillium italicum		15: bovine	f(17-31)	[114]
		6: bovine	f(20-25)	[114]
Penicillium notatum	Bovine (R)		f(17-41)	[203]
Penicillium pinophilum	Bovine		f(17-41)	[204]
Penicillium vermiculathm	Bovine		f(17-41)	[204]

Phialophora verrucosa		Bovine		f(17-41)	[203]
Rhizopus oryzae		Bovine (R)		f(17-41)	[203, 204]
Sporothrix schenckii		Bovine		f(17-41)	[203]
Trichophyton mentagrophytes		Bovine (some R)		f(17-41)	[203, 204]
Trichophyton rubrum		Bovine		f(17-41)	[203, 204]
Trichophyton tonsurans		Bovine		f(17-41)	[203]
Trichophyton shoenleinii		Bovine (R)		f(17-41)	[203]
Trichophyton violaceum		Bovine (some R)		f(17-41)	[203]
**Parasites**					
Babesia caballi	Bovine			/	[208]
Giardia intestinalis	Bovine			/	[212]
		Bovine		f(17-41)	[212]
			Bovine	f(268-284)	[212]
	Chimera: bovine			f(17-30)-(265-284)	[212]
Giardia lamblia	Bovine			/	[210, 211, 213]
		Bovine		f(17-41)	[210, 211, 213]
Toxsoplasma gondii	Bovine (R)			/	[225, 226]
		Bovine		f(17-41)	[225, 228, 229]

[1] Lactoferrin-derived peptides are named as in original articles; [2]sequence of peptide fraction correspond to the sequence of the source lactoferrin

*Due to the poor stability of the TNGESTADWAKN f(552-563) peptide, a modified sequence (NGESSADWAKN) was designed where the first threonine was removed and the other one was replaced by serine.
